# Data sharing in clinical trials - practical guidance on anonymising trial datasets

**DOI:** 10.1186/1745-6215-16-S2-O66

**Published:** 2015-11-16

**Authors:** Christopher Tuck, Steff Lewis, Garry Milne, Sandra Eldridge, Neil Wright

**Affiliations:** 1 University of Edinburgh, Edinburgh, UK; 2 Queen Mary University of London, London, UK

## 

There is an increasing demand by non-commercial funders that trialists should provide access to trial data once the primary analysis is completed [1]. This has to take into account concerns about identifying individual trial participants, and the legal and regulatory requirements.

Using the good practice guidelines on data sharing laid out by the work funded by the MRC Network of Hubs for Trials Methodology Research [2], a collaborative effort between the University of Edinburgh and Queen Mary University of London, as part of the Asthma UK Centre for Applied Research, is devising processes and mechanisms for user access control, anonymisation and sharing of clinical trial data.

As part of this, we have anonymised a dataset from a recently completed trial [3]. Using this example, we will present practical guidance on how to anonymise a dataset, and describe rules that could be used on other trial datasets. We will describe how these might differ if the trial was to be made freely available to all, or if the data could only be accessed with specific permission and data usage agreements in place.
